# Robotic resection of ectopic mediastinal parathyroid adenoma with intraoperative parathyroid hormone monitoring: a case report

**DOI:** 10.1186/s13019-022-01935-2

**Published:** 2022-08-20

**Authors:** Yoshihito Iijima, Masahito Ishikawa, Shun Iwai, Aika Yamagata, Kazuhiro Kishimoto, Nozomu Motono, Hidetaka Uramoto

**Affiliations:** 1 Department of Thoracic Surgery, Kanazawa Medical University, 1-1 Daigaku, Uchinada-machi, Kahoku-gun, Ishikawa 920-0293 Japan; 2 Department of Head and Neck Surgery, Kanazawa Medical University, Kahoku-gun, Ishikawa Japan

**Keywords:** Ectopic mediastinal parathyroid adenoma, Parathyroid hormone, Intraoperative monitoring, Robotic resection

## Abstract

**Background:**

Primary hyperparathyroidism is a disease caused by the secretion of excess parathyroid hormone (PTH) owing to the enlargement of the parathyroid gland. Ectopic parathyroid glands exist in the mediastinum in approximately 1–2% of cases, which is relatively rare. Intraoperative monitoring of serum PTH level is important to assess whether the source of hyperparathyroidism has been eliminated.

**Case presentation:**

A 53-year-old asymptomatic woman was diagnosed with ectopic mediastinal parathyroid adenoma. A three-port robotic partial resection of the thymus containing the tumor was attempted, but bleeding from a swollen pericardial diaphragmatic vein led to the addition of an assist port along the way. The PTH level was measured intraoperatively. After confirming that the 15-min PTH level after removal of the tumor was less than 50% of the baseline value, the operation was completed. The tumor was positive for PTH and was diagnosed as an ectopic mediastinal parathyroid adenoma. Some small ectopic parathyroid gland tissues were observed in other parts of the thymic tissue. Serum calcium and PTH levels decreased and normalized.

**Conclusions:**

We report the usefulness of robotic resection for ectopic mediastinal parathyroid adenoma with PTH monitoring. However, histopathologically, small parathyroid gland tissues may remain in the surrounding thymus. Hence, we believe that a strict follow-up is required for parathyroid function in the future.

## Background

Primary hyperparathyroidism (PHPT) is caused by the secretion of excess parathyroid hormone (PTH) owing to the enlargement of the parathyroid gland. It is classified as follows according to the disease type: asymptomatic biochemical type, calculus type with urinary tract stones, and bone type with pathological fracture. Approximately 85% of PHPT cases are due to adenomas [1]. There are 300 to 400 new cases of PHPT per year in Japan, with a male–female ratio of approximately 1:2 [2]. In some cases, ectopic parathyroid glands and excess glands may be present. Parathyroid adenomas are often located near the thyroid gland in the neck; however, ectopic parathyroid adenomas may be present. Ectopic parathyroid adenoma is reported in 15–20% cases and ectopic mediastinal parathyroid adenoma (EMPA), which is necessary for a thoracic approach, is reported in 1–2%, which is relatively rare [3, 4]. The half-life of PTH in the circulatory system is less than 5 min [5], therefore, serum PTH monitoring can be used intraoperatively to assess whether the source of hyperparathyroidism has been eliminated [6, 7]. Herein, we report the usefulness of robotic EMPA resection with PTH monitoring.

## Case presentation

**Fig. 1:**
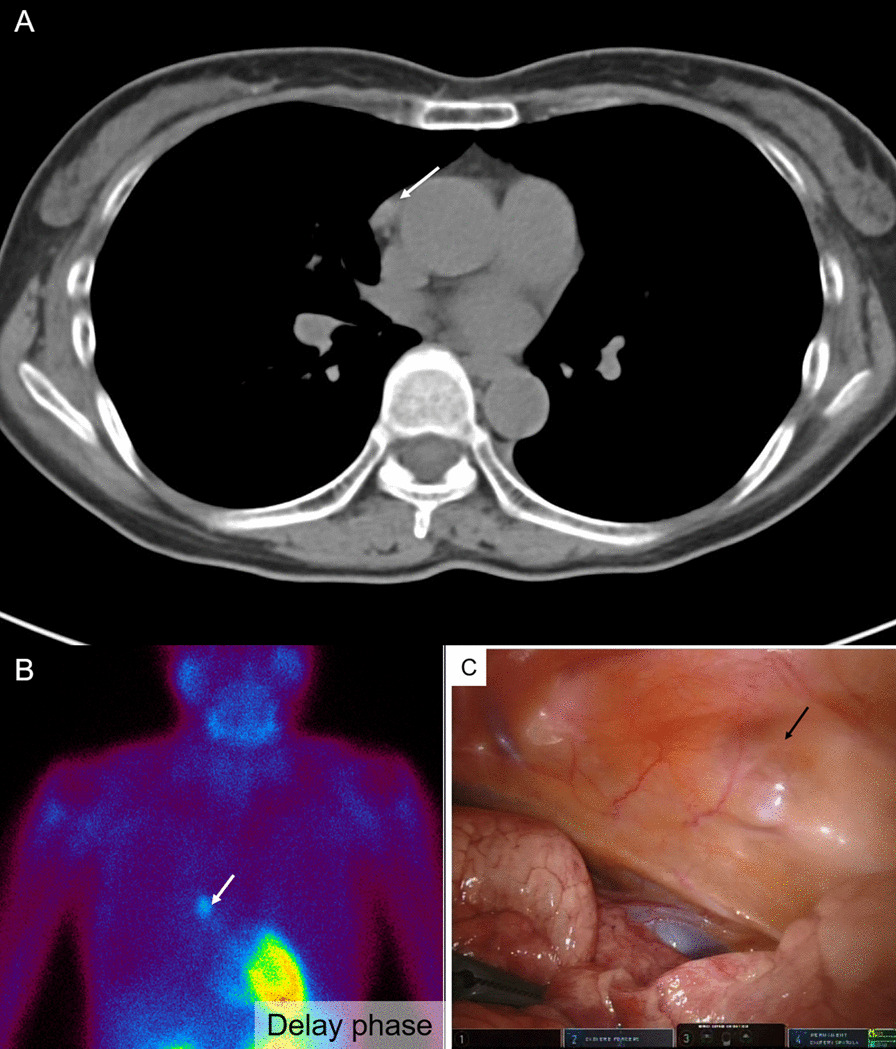
Computed tomography (CT) imaging and Technetium-99m-methoxy-isobutyl-isonitrile (99mTc-MIBI) scintigraphy. **A** Chest CT showed anterior mediastinal nodule (white arrow). **B** 99mTc-MIBI scintigraphy showed no accumulation at delay phase in the thyroid gland, but accumulation in the anterior mediastinal nodule (white arrow). **C** Intraoperative findings. The location of the tumor (black arrow) was immediately identifiable

A 53-year-old asymptomatic woman presented with hypercalcemia during a medical examination and visited the Department of Endocrinology and Metabolism at our institution. She had a serum calcium level of 11.7 mg/dL, a urinary calcium/creatinine ratio of 0.39, and an intact PTH level of 268 pg/mL. She was suspected to have PHPT. A fracture of the vertebral bone was not detected, but the T-score was -3.8, indicating osteoporosis. The creatinine clearance was 79.9 mL/min and the urinary calcium excretion was 409 mg/day. Neck ultrasonography (US) showed a solid thyroid nodule measuring approximately 5 mm and no swelling of the parathyroid gland was observed. Chest computed tomography (CT) showed an anterior mediastinal nodule (Fig. 1A). Abdominal CT did not show urinary stones or calcification of the kidney. Technetium-99m-methoxy-isobutyl-isonitrile (^99m^Tc-MIBI) scintigraphy showed no accumulation in the delay phase in the thyroid gland, but accumulation was observed both in the early and delayed phases in the anterior mediastinal nodule (Fig. 1B), and the diagnosis of EMPA was established.

**Fig. 2:**
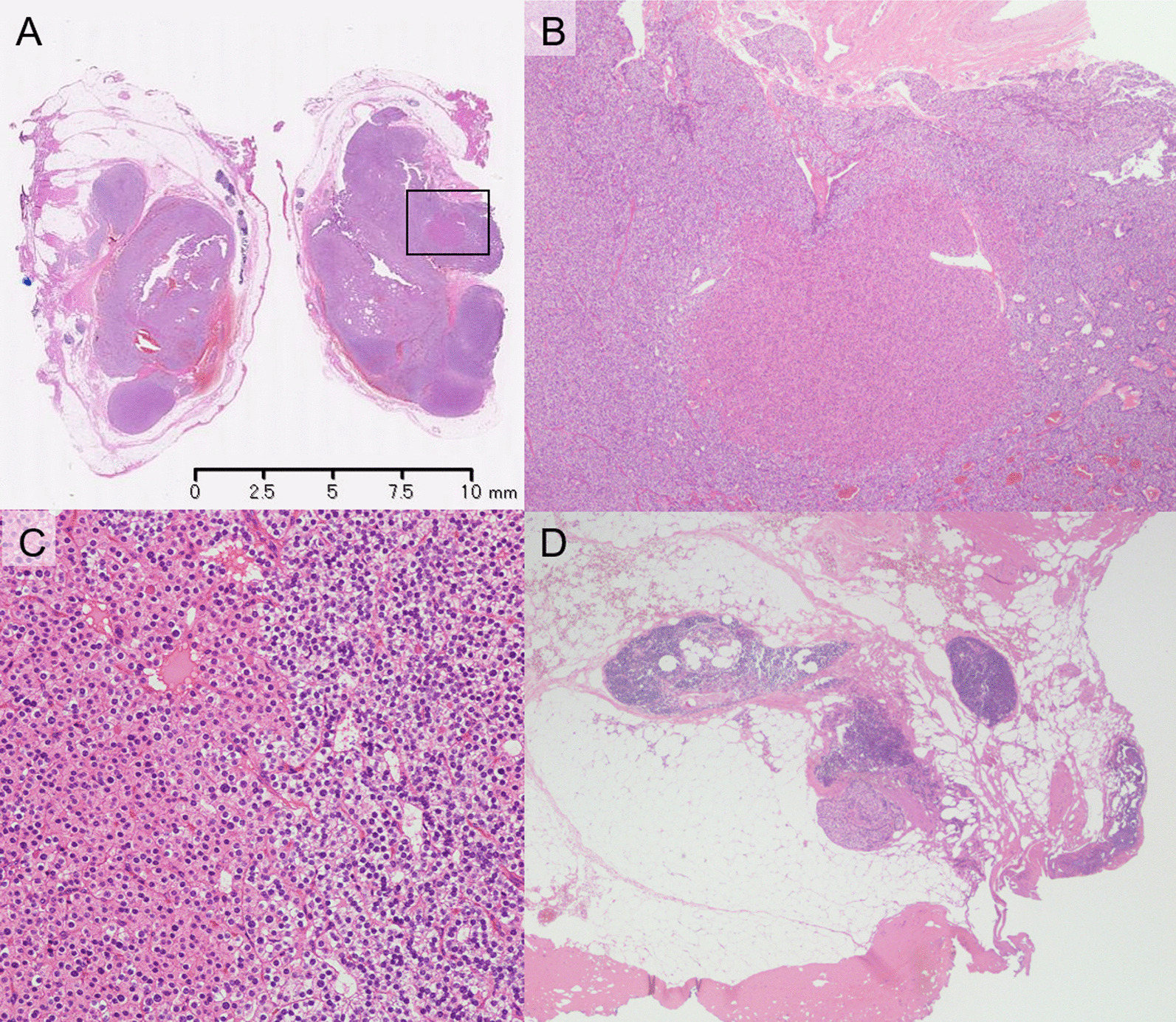
Pathorogical findings. **A** The tumors were well circumscribed tumor, 12 mm in size, and were covered with capsules. (Hematoxylin–Eosin (H.E.) stain, loupe magnification) (**B**, **C**) the cubic cells, with clear and eosinophilic cytoplasm and uniform nuclei, arranged in solid sheets. (**B** H.E. stain, × 4 magnification, the black rectangle in (**A**) is the same area as shown in (**B**). **C** H.E. stain, × 20 magnification), **D** some small parathyroid gland tissues were observed in the other part of thymic tissue (H.E. stain, × 4 magnification)

We decided to remove the EMPA through collaborative consultation between the Department of Endocrinology and Metabolism, Department of Head and Neck Surgery, and Department of Thoracic Surgery. As per the discussions, it was decided to perform EMPA resection while performing intraoperative PTH monitoring. Cinacalcet hydrochloride was administered orally at a dose of 50 mg/day before surgery. A three-port robotic partial resection of the thymus with the EMPA was attempted (Fig. 1C), but bleeding from a swollen pericardial diaphragmatic vein led to the addition of an assist port along the way. Intact PTH value was measured after the induction of anesthesia, immediately before removal of the adenoma, and 5 min, 10 min, 15 min, and 60 min after removal. The intact PTH levels were 326.1 pg/mL, 975.0 pg/mL, 252.0 pg/mL, 195.7 pg/mL, 155.1 pg/mL and 57.2 pg/mL, respectively. After confirming that the 15-min intact PTH value after removal of the tumor was less than 50% of the baseline value, the operation was completed without additional resection. Since the intact PTH value was sufficiently low, we decided to omit confirming the absence of residual glandular tissue using methylene blue (MB) staining method. The operation time was 76 min, the console time was 16 min, and the amount of bleeding was less. The tumors were well circumscribed, 12 mm in size, and were covered with capsules (Fig. 2A). Histopathologically, the cubic cells, with clear and eosinophilic cytoplasm and uniform nuclei, were arranged in solid sheets (Fig. 2B, C). Mitoses were absent, and there was no capsule infiltration or failure. However, some small parathyroid gland tissues were observed in the other parts of the thymic tissue (Fig. 2D); therefore, it was suspected that microscopic ectopic parathyroid gland tissue may have remained in the thymus. Immunohistochemically, the tumor was positive for PTH-rerated protein and was diagnosed as an EMPA. On postoperative day (POD) 1, the serum calcium level decreased to 8.8 mg/dL and the intact PTH level decreased to 25 pg/mL. Oral administration of calcium lactate and calcitriol was started, and the dose of calcium lactate was gradually decreased, and it was discontinued on the POD 13 as the serum calcium level stabilized. On POD 23, she was discharged from the hospital and is currently an outpatient.

## Discussion and conclusions

Resection of EMPA in the upper mediastinum using a transcervical approach with or without partial sternotomy may be common, but resection by video-assisted thoracic surgery (VATS) is common in the deep mediastinum. VATS is an approach with less surgical trauma, decreased associated morbidity, shorter hospital stays, and superior cosmetic results. In recent years, several cases of robot-assisted thoracic surgery (RATS) for EMPA have been reported [8–11]. Ward et al. reported that robot-assisted complete thymectomy for EMPA, which provides excellent visualization of the mediastinum, is effective in reducing PTH and calcium levels and is safe with no morbidity or mortality [8].

Prior to the surgical removal of an EMPA, the surgeon should attempt to understand the status of the patient's cervical parathyroid glands [1]. Parathyroid tissue function has profound clinical implications, and leaving a patient with loss of parathyroid function should be avoided when possible. Autografting of hyperfunctional parathyroid tissue may be necessary. Therefore, thoracic surgeons should be aware of this scenario, and if necessary, an endocrine surgeon should be included in the management of the patient [1]. ^99m^Tc-MIBI and neck US are the initial imaging modalities for patients with PHPT. Regardless of the imaging modality used for localization, the surgeon must exercise caution because no examination is 100% accurate in diagnosing a single adenoma as the cause of PHPT [12]. In this case, ^99m^Tc-MIBI showed accumulation in the anterior mediastinal nodule, which appeared as a single solid lesion. The EMPA was removed with the surrounding thymus without damaging the capsule; however, there were small parathyroid gland tissues in the surrounding thymus. This indicates that excision of the main tumor alone may not normalize parathyroid function. In fact, even the high-volume center has an operative failure rate of 13–22% [6, 13]. In addition, a recurrence of secondary hyperparathyroidism, especially in patients undergoing dialysis patients, is possible [14]. Therefore, it is extremely important to monitor the PTH levels during surgery.

There is a report in the field of head and neck surgery that dissemination occurs during parathyroidectomy [1, 15]. It is important not only to handle the tumor gently, but also to know the exact location of the tumor. In this case, the patient was a lean woman with little fat in the mediastinum, and the location of the tumor was immediately identifiable. However, most EMPAs are buried in the thymus and adipose tissue, and it may be difficult to identify lesions during surgery even after careful examination before surgery. The intraoperative radio-guide method in which ^99m^Tc-MIBI is intravenously injected at the time of surgery [16, 17] and a staining method in which MB is intravenously injected [18, 19] have been reported as identification methods. Moreover, in recent years, near-infrared fluorescence imaging using indocyanine green has been applied to identify the parathyroid gland intraoperatively [20]. The radio-guide method is not suitable for EMPAs in the deep mediastinum because of the effect of ^99m^Tc-MIBI uptake into the myocardium and great vessels [16]. Pseudohypoxemia is a problem in anesthesia management using the MB staining method, but it can be solved using near-infrared spectroscopy [21].

In conclusion, we successfully resected an EMPA using RATS with intraoperative PTH monitoring. However, histopathologically, small parathyroid gland tissues may remain in the surrounding thymus. Hence, we believe that a strict follow-up is required to monitor parathyroid function following RATS.

## Data Availability

All data generated or analyzed during this study are included in this published article.

## Abbreviations

PHPT: Primary hyperparathyroidism
PTH: Parathyroid hormone
EMPA: Ectopic mediastinal parathyroid adenoma
US: Ultrasonography
CT: Computed tomography
^99m^Tc-MIBI: Technetium-99m-methoxy-isobutyl-isonitrile
MB: Methylene blue
VATS: Video-assisted thoracic surgery
RATS: Robot-assisted thoracic surgery
POD: Postoperative days
